# Ten simple rules for reading a scientific paper

**DOI:** 10.1371/journal.pcbi.1008032

**Published:** 2020-07-30

**Authors:** Maureen A. Carey, Kevin L. Steiner, William A. Petri

**Affiliations:** Division of Infectious Diseases and International Health, Department of Medicine, University of Virginia School of Medicine, Charlottesville, Virginia, United States of America; Dassault Systemes BIOVIA, UNITED STATES

## Introduction

“There is no problem that a library card can't solve” according to author Eleanor Brown [1]. This advice is sound, probably for both life and science, but even the best tool (like the library) is most effective when accompanied by instructions and a basic understanding of how and when to use it.

For many budding scientists, the first day in a new lab setting often involves a stack of papers, an email full of links to pertinent articles, or some promise of a richer understanding so long as one reads enough of the scientific literature. However, the purpose and approach to reading a scientific article is unlike that of reading a news story, novel, or even a textbook and can initially seem unapproachable. Having good habits for reading scientific literature is key to setting oneself up for success, identifying new research questions, and filling in the gaps in one’s current understanding; developing these good habits is the first crucial step.

Advice typically centers around two main tips: read actively and read often. However, active reading, or reading with an intent to understand, is both a learned skill and a level of effort. Although there is no one best way to do this, we present 10 simple rules, relevant to novices and seasoned scientists alike, to teach our strategy for active reading based on our experience as readers and as mentors of undergraduate and graduate researchers, medical students, fellows, and early career faculty. Rules 1–5 are big picture recommendations. Rules 6–8 relate to philosophy of reading. Rules 9–10 guide the “now what?” questions one should ask after reading and how to integrate what was learned into one’s own science.

## Rule 1: Pick your reading goal

What you want to get out of an article should influence your approach to reading it. **Table 1** includes a handful of example intentions and how you might prioritize different parts of the same article differently based on your goals as a reader.

**Table 1:** Reading intentions and how it might influence your approach.

Examples	Intention	Priorities
1	You are new to reading scientific papers.^1^	For each panel of each figure, focus particularly on the questions outlined in Rule 3.
2	You are entering a new field and want to learn what is important in that field.	Focus on the beginning (motivation presented in the introduction) and the end (next steps presented in the conclusion).
3	You receive automated alerts to notify you of the latest publication from a particular author whose work inspires you; you are hoping to work with them for the next phase of your research career and want to know what they are involved in.	Skim the entire work, thinking about how it fits into the author’s broader publication history.
4	You receive automated alerts to notify you of the latest publication containing a set of keywords because you want to be aware of new ways a technique is being applied or the new developments in a particular topic or research area.	Focus on what was done in the methods and the motivation for the approach taken; this is often presented in the introduction.
5	You were asked to review an article prior to publication to evaluate the quality of work or to present in a journal club.^2^	Same as example 1. Also, do the data support the interpretations? What alternative explanations exist? Are the data presented in a logical way so that many researchers would be able to understand? If the research is about a controversial topic, do the author(s) appropriately present the conflict and avoid letting their own biases influence the interpretation?

^1^ Yay! Welcome!

^2^ A journal club is when a group of scientists get together to discuss a paper. Usually one person leads the discussion and presents all of the data. The group discusses their own interpretations and the authors’ interpretation.

## Rule 2: Understand the author’s goal

In written communication, the reader and the writer are equally important. Both influence the final outcome: in this case, your scientific understanding! After identifying your goal, think about the author’s goal for sharing this project. This will help you interpret the data and understand the author’s interpretation of the data. However, this requires some understanding of who the author(s) are (e.g., what are their scientific interests?), the scientific field in which they work (e.g., what techniques are available in this field?), and how this paper fits into the author’s research (e.g., is this work building on an author’s longstanding project or controversial idea?). This information may be hard to glean without experience and a history of reading. But don’t let this be a discouragement to starting the process; it is by the act of reading that this experience is gained!

A good step toward understanding the goal of the author(s) is to ask yourself: What kind of article is this? Journals publish different types of articles, including methods, review, commentary, resources, and research articles as well as other types that are specific to a particular journal or groups of journals. These article types have different formatting requirements and expectations for content. Knowing the article type will help guide your evaluation of the information presented. Is the article a methods paper, presenting a new technique? Is the article a review article, intended to summarize a field or problem? Is it a commentary, intended to take a stand on a controversy or give a big picture perspective on a problem? Is it a resource article, presenting a new tool or data set for others to use? Is it a research article, written to present new data and the authors’ interpretation of those data? The type of paper, and its intended purpose, will get you on your way to understanding the author’s goal.

## Rule 3: Ask six questions

When reading, ask yourself: (1) What do the author(s) want to know (motivation)? (2) What did they do (approach/methods)? (3) Why was it done that way (context within the field)? (4) What do the results show (figures and data tables)? (5) How did the author(s) interpret the results (interpretation/discussion)? (6) What should be done next? (Regarding this last question, the author(s) may provide some suggestions in the discussion, but the key is to ask yourself what you think should come next.)

Each of these questions can and should be asked about the complete work as well as each table, figure, or experiment within the paper. Early on, it can take a long time to read one article front to back, and this can be intimidating. Break down your understanding of each section of the work with these questions to make the effort more manageable.

## Rule 4: Unpack each figure and table

Scientists write original research papers primarily to present new data that may change or reinforce the collective knowledge of a field. Therefore, the most important parts of this type of scientific paper are the data. Some people like to scrutinize the figures and tables (including legends) before reading any of the “main text”: because all of the important information should be obtained through the data. Others prefer to read through the results section while sequentially examining the figures and tables as they are addressed in the text. There is no correct or incorrect approach: Try both to see what works best for you. The key is making sure that one understands the presented data and how it was obtained.

For each figure, work to understand each x- and y-axes, color scheme, statistical approach (if one was used), and why the particular plotting approach was used. For each table, identify what experimental groups and variables are presented. Identify what is shown and how the data were collected. This is typically summarized in the legend or caption but often requires digging deeper into the methods: Do not be afraid to refer back to the methods section frequently to ensure a full understanding of how the presented data were obtained. Again, ask the questions in Rule 3 for each figure or panel and conclude with articulating the “take home” message.

## Rule 5: Understand the formatting intentions

Just like the overall intent of the article (discussed in Rule 2), the intent of each section within a research article can guide your interpretation. Some sections are intended to be written as objective descriptions of the data (i.e., the Results section), whereas other sections are intended to present the author’s interpretation of the data. Remember though that even “objective” sections are written by and, therefore, influenced by the authors interpretations. Check out **Table 2** to understand the intent of each section of a research article. When reading a specific paper, you can also refer to the journal’s website to understand the formatting intentions. The “For Authors” section of a website will have some nitty gritty information that is less relevant for the reader (like word counts) but will also summarize what the journal editors expect in each section. This will help to familiarize you with the goal of each article section.

**Table 2:** The structure of a primary research article.

Section	Content
Title	The “take home” message of the entire project, according to the authors.
Author list	These people made significant scientific contributions to the project. Fields differ in the standard practice for ordering authors. For example, as a general rule for biomedical sciences, the first author led the project’s implementation, and the last author was the primary supervisor to the project.
Abstract	A brief overview of the research question, approach, results, and interpretation. This is the road map or elevator pitch for an article.
Introduction	Several paragraphs (or less) to present the research question and why it is important. A newcomer to the field should get a crash course in the field from this section.
Methods	What was done? How was it done? Ideally, one should be able to recreate a project by reading the methods. In reality, the methods are often overly condensed. Sometimes greater detail is provided within a “Supplemental” section available online (see below).
Results	What was found? Paragraphs often begin with a statement like this: “To do X, we used approach Y to measure Z.” The results should be objective observations.
Figures, tables, legends, and captions	The data are presented in figures and tables. Legends and captions provide necessary information like abbreviations, summaries of methods, and clarifications.
Discussion	What do the results mean and how do they relate to previous findings in the literature? This is the perspective of the author(s) on the results and their ideas on what might be appropriate next steps. Often it may describe some (often not all!) strengths and limitations of the study: Pay attention to this self-reflection of the author(s) and consider whether you agree or would add to their ideas.
Conclusion	A brief summary of the implications of the results.
References	A list of previously published papers, datasets, or databases that were essential for the implementation of this project or interpretation of data. This section may be a valuable resource listing important papers within the field that are worth reading as well.
Supplemental material	Any additional methods, results, or information necessary to support the results or interpretations presented in the discussion.
Supplemental data	Essential datasets that are too large or cumbersome to include in the paper. Especially for papers that include “big data” (like sequencing or modeling results), this is often where the real, raw data is presented.

Research articles typically contain each of these sections, although sometimes the “results” and “discussion” sections (or “discussion” and “conclusion” sections) are merged into one section. Additional sections may be included, based on request of the journal or the author(s). Keep in mind: If it was included, someone thought it was important for you to read.

## Rule 6: Be critical

Published papers are not truths etched in stone. Published papers in high impact journals are not truths etched in stone. Published papers by bigwigs in the field are not truths etched in stone. Published papers that seem to agree with your own hypothesis or data are not etched in stone. Published papers that seem to refute your hypothesis or data are not etched in stone.

Science is a never-ending work in progress, and it is essential that the reader pushes back against the author’s interpretation to test the strength of their conclusions. Everyone has their own perspective and may interpret the same data in different ways. Mistakes are sometimes published, but more often these apparent errors are due to other factors such as limitations of a methodology and other limits to generalizability (selection bias, unaddressed, or unappreciated confounders). When reading a paper, it is important to consider if these factors are pertinent.

Critical thinking is a tough skill to learn but ultimately boils down to evaluating data while minimizing biases. Ask yourself: Are there other, equally likely, explanations for what is observed? In addition to paying close attention to potential biases of the study or author(s), a reader should also be alert to one’s own preceding perspective (and biases). Take time to ask oneself: Do I find this paper compelling because it affirms something I already think (or wish) is true? Or am I discounting their findings because it differs from what I expect or from my own work?

The phenomenon of a self-fulfilling prophecy, or expectancy, is well studied in the psychology literature [2] and is why many studies are conducted in a “blinded” manner [3]. It refers to the idea that a person may assume something to be true and their resultant behavior aligns to make it true. In other words, as humans and scientists, we often find exactly what we are looking for. A scientist may only test their hypotheses and fail to evaluate alternative hypotheses; perhaps, a scientist may not be aware of alternative, less biased ways to test her or his hypothesis that are typically used in different fields. Individuals with different life, academic, and work experiences may think of several alternative hypotheses, all equally supported by the data.

## Rule 7: Be kind

The author(s) are human too. So, whenever possible, give them the benefit of the doubt. An author may write a phrase differently than you would, forcing you to reread the sentence to understand it. Someone in your field may neglect to cite your paper because of a reference count limit. A figure panel may be misreferenced as Supplemental Fig 3E when it is obviously Supplemental Fig 4E. While these things may be frustrating, none are an indication that the quality of work is poor. Try to avoid letting these minor things influence your evaluation and interpretation of the work.

Similarly, if you intend to share your critique with others, be extra kind. An author (especially the lead author) may invest years of their time into a single paper. Hearing a kindly phrased critique can be difficult but constructive. Hearing a rude, brusque, or mean-spirited critique can be heartbreaking, especially for young scientists or those seeking to establish their place within a field and who may worry that they do not belong.

## Rule 8: Be ready to go the extra mile

To truly understand a scientific work, you often will need to look up a term, dig into the supplemental materials, or read one or more of the cited references. This process takes time. Some advisors recommend reading an article three times: The first time, simply read without the pressure of understanding or critiquing the work. For the second time, aim to understand the paper. For the third read through, take notes.

Some people engage with a paper by printing it out and writing all over it. The reader might write question marks in the margins to mark parts (s)he wants to return to, circle unfamiliar terms (and then actually look them up!), highlight or underline important statements, and draw arrows linking figures and the corresponding interpretation in the discussion. Not everyone needs a paper copy to engage in the reading process but, whatever your version of “printing it out” is, do it.

## Rule 9: Talk about it

Talking about an article in a journal club or more informal environment forces active reading and participation with the material. Studies show that teaching is one of the best ways to learn and that teachers learn the material even better as the teaching task becomes more complex [4–5]; anecdotally, such observations inspired the phrase “to teach is to learn twice.”

Beyond formal settings such as journal clubs, lab meetings, and academic classes, discuss papers with your peers, mentors, and colleagues in person or electronically. Twitter and other social media platforms have become excellent resources for discussing papers with other scientists, the public or your nonscientist friends, or even the paper’s author(s). Describing a paper can be done at multiple levels and your description can contain all of the scientific details, only the big picture summary, or perhaps the implications for the average person in your community. All of these descriptions will solidify your understanding, while highlighting gaps in your knowledge and informing those around you.

## Rule 10: Build on it

One approach we like to use for communicating how we build on the scientific literature is by starting research presentations with an image depicting a wall of Lego bricks. Each brick is labeled with the reference for a paper, and the wall highlights the body of literature on which the work is built. We describe the work and conclusions of each paper represented by a labeled brick and discuss each brick and the wall as a whole. The top brick on the wall is left blank: We aspire to build on this work and label this brick with our own work. We then delve into our own research, discoveries, and the conclusions it inspires. We finish our presentations with the image of the Legos and summarize our presentation on that empty brick.

Whether you are reading an article to understand a new topic area or to move a research project forward, effective learning requires that you integrate knowledge from multiple sources (“click” those Lego bricks together) and build upwards. Leveraging published work will enable you to build a stronger and taller structure. The first row of bricks is more stable once a second row is assembled on top of it and so on and so forth. Moreover, the Lego construction will become taller and larger if you build upon the work of others, rather than using only your own bricks.

Build on the article you read by thinking about how it connects to ideas described in other papers and within own work, implementing a technique in your own research, or attempting to challenge or support the hypothesis of the author(s) with a more extensive literature review. Integrate the techniques and scientific conclusions learned from an article into your own research or perspective in the classroom or research lab. You may find that this process strengthens your understanding, leads you toward new and unexpected interests or research questions, or returns you back to the original article with new questions and critiques of the work. All of these experiences are part of the “active reading”: process and are signs of a successful reading experience.

In summary, practice these rules to learn how to read a scientific article, keeping in mind that this process will get easier (and faster) with experience. We are firm believers that an hour in the library will save a week at the bench; this diligent practice will ultimately make you both a more knowledgeable and productive scientist. As you develop the skills to read an article, try to also foster good reading and learning habits for yourself (recommendations here: [6] and [7], respectively) and in others. Good luck and happy reading!

## References

[1] Brown E. The Weird Sisters. G. P. Putnam’s Sons; 2011.

[2] Kirsch I. Response expectancy theory and application: A decennial review. Appl Prev Psychol. 1997 3 1;6(2):69–79.

[3] Karanicolas PJ, Farrokhyar F, Bhandari M. Practical tips for surgical research: blinding: who, what, when, why, how? Can J Surg. 2010 10;53(5):345–8. 20858381 PMC2947122

[4] Fiorella L, Mayer RE. The relative benefits of learning by teaching and teaching expectancy. Contemp Educ Psychol. 2013 10 1;38(4):281–8.

[5] Koh AWL, Lee SC, Lim SWH. The learning benefits of teaching: A retrieval practice hypothesis. Appl Cogn Psychol. 2018 5 15;32(3):401–10.

[6] Méndez M. Ten simple rules for developing good reading habits during graduate school and beyond. PLoS Comput Biol. 2018 10;14(10):e1006467 10.1371/journal.pcbi.1006467 30307941 PMC6181267

[7] Erren TC, Slanger TE, Groß JV, Bourne PE, Cullen P. Ten simple rules for lifelong learning, according to Hamming. PLoS Comput Biol. 2015 2;11(2):e1004020 10.1371/journal.pcbi.1004020 25719205 PMC4342007

